# Small ring has big potential: insights into extrachromosomal DNA in cancer

**DOI:** 10.1186/s12935-021-01936-6

**Published:** 2021-04-26

**Authors:** Yihao Wang, Rui Huang, Guopei Zheng, Jianfeng Shen

**Affiliations:** 1 Department of Ophthalmology, Ninth People’s Hospital, Shanghai JiaoTong University School of Medicine, Shanghai, 200025 China; 2 Shanghai Key Laboratory of Orbital Diseases and Ocular Oncology, Shanghai, 200025 China

**Keywords:** EcDNA, Oncogene amplification, Chromosomal rearrangement, Epigenetic modification, Tumor heterogeneity

## Abstract

Recent technical advances have led to the discovery of novel functions of extrachromosomal DNA (ecDNA) in multiple cancer types. Studies have revealed that cancer-associated ecDNA shows a unique circular shape and contains oncogenes that are more frequently amplified than that in linear chromatin DNA. Importantly, the ecDNA-mediated amplification of oncogenes was frequently found in most cancers but rare in normal tissues. Multiple reports have shown that ecDNA has a profound impact on oncogene activation, genomic instability, drug sensitivity, tumor heterogeneity and tumor immunology, therefore may offer the potential for cancer diagnosis and therapeutics. Nevertheless, the underlying mechanisms and future applications of ecDNA remain to be determined. In this review, we summarize the basic concepts, biological functions and molecular mechanisms of ecDNA. We also provide novel insights into the fundamental role of ecDNA in cancer.

## Introduction

Extrachromosomal DNA (ecDNA) is a particular type of DNA molecule outside of the chromosome that is usually 1–3 Mb in length [1]. EcDNA does not contain centromeres or telomeres, but it has regulatory regions that control the expression of the encoded genes [2, 3]. Studies have shown that ecDNA accounts for nearly 30% of all DNA particles outside of the chromosome [4, 5]. In addition, there are two major categories of extrachromosomal DNA particles that differ from ecDNA in sequence length: (1) extrachromosomal small circular DNA (eccDNA) and (2) ring- or neochromosome (Table 1) [6–9]. eccDNA is a double-stranded circular molecule less than 1 Mb in length that consists of multiple copies of genome-originated repetitive non-coding DNA sequences and telomeric circles (e.g. small polydispersed circular DNA and microDNA) [10]. In contrast to ecDNA that is rarely seen in normal cells, eccDNA is present in both normal cells and cancer cells, and eccDNA may promote tumorigenesis through the selection of telomere extension and modulation of genomic instability [10, 11]. In ring-chromosomes, the ends of the DNA sequence are fused together to form a ring shape [12]. Neochromosomes contain centromere and telomere sequences, with a typical sequence length of 30–600 Mb [7, 8]. Neochromosomes have been shown to contain high copy numbers of oncogenes and can be created through chromothripsis [9].

**Table 1:** Characteristics of ecDNA, eccDNA, neo and ring chromosome

	Size	Single/double strand	Sequence feature	Definition	Origination	Refs.
ecDNA	1-3 Mb, visible under microscope	Double	Oncogene amplification, regulatory regions, no centromeres or telomeres	Extrachromosomal DNA (double minutes)	BFB cycle, translocation-deletion-amplification, episome and chromothripsis	[1, 3]
eccDNA	< 1 Mb. Invisible under microscope	Single or double	Small regulatory RNA	Extrachromosomal small circular DNA	Telomere circle, spcDNA, miDNA, episome	[93, 94]
Neo chromosome	30–600 Mb, visible under microscope	Double	Contains centromere or telomere	Structurally abnormal chromosome	Chromothripsis and BFB cycles with telomere aggregation	[7, 9]
Ring chromosome	1.4–7.3 cms. Visible under microscope	Double	Circular or linear form, contains centromere and telomere	Breaks of telomeric ends, end-to-end fusion of the centric chromosome	End joining of DNA double-strand breaks, telomere_subtelomere junction, or rearrangement	[6]

Recent findings have revealed the essential roles of ecDNA in cancer [1, 2, 12–14]. ecDNA is widely expressed in multiple types of cancers, including highly aggressive glioblastoma and sarcoma, but not in normal tissues [2, 15]. The presence of ecDNA is also associated with the rapid amplification of oncogenes and elevated intra-tumoral heterogeneity [15]. Moreover, the lack of centromeres in ecDNA leads to the discordant inheritance of ecDNA elements during mitosis, contributing to the hyper-activity of oncogene expression. These features of ecDNA endow cancer cells with the ability of quick adaptation toward the microenvironment, therefore promoting intra-tumoral heterogeneity [1, 16]. The study of ecDNA in cancer is still in its infancy. In this review, we summarize the recent findings of ecDNA regarding the structure, biogenesis, function and therapeutic potentials in cancer.

## The biological features of ecDNA

### Structure

Early attempts to uncover the structure of ecDNA were limited due to technical obstacles. Recent advances in next-generation sequencing technologies (e.g. whole genome sequencing) and computational analytical approaches have led to the discovery that ecDNA presents in a circular shape and can replicate independently outside of chromosomes [2, 16, 17]. In addition, ecDNA contains not only complete genes, but also regulatory elements such as upstream promoters and enhancers [2, 3]. Importantly, rewiring of adjacent enhancers along with endogenous enhancers was observed in ecDNA [3]. In certain circumstances, ecDNA can incorporate DNA segments from different chromosomes to form chimeric sequences, which may subsequently “invade” and re-integrate into other chromosomes to generate novel DNA sequences [2]. In comparison with linear DNA, ecDNA has a highly accessible chromatin state and significantly higher levels of H3K27ac, a well-established histone marker for super enhancers [2, 3]. These structural characteristics of ecDNA markedly elevate the expression levels of oncogenes in ecDNA and affect chromatin rearrangement to promote intra-tumoral heterogeneity [16]. Verhaak et al. analyzed circular DNA data from 3,212 patients across a variety of cancer types. The association between oncogene amplification and ecDNA structure was observed, however such association could not be applied to the breakpoint; and the distributions of oncogene amplicons were highly nonrandom [15]. These results demonstrate that not only the inherited “genetic code,” but also the topology and three-dimensional chromatin landscape play critical roles in maintaining proper function of the cancer genome [1, 15, 18].

### Biogenesis

**Fig. 1:**
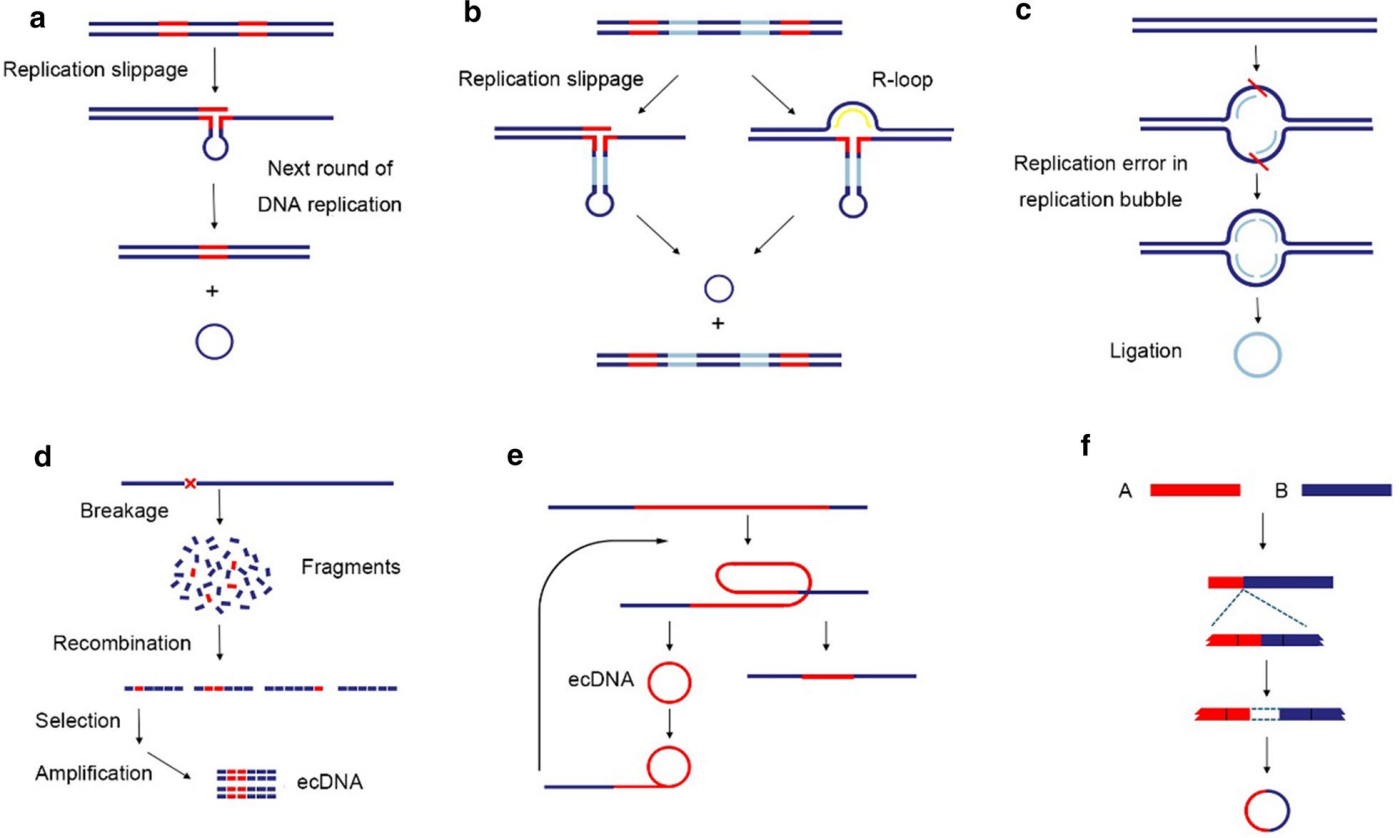
Models of ecDNA formation. **a** Replication slippage model 1: ecDNA can be generated from replication slippage where DNA polymerase replicates DNA at wrong direction and creates a loop on the template strand. The loop is then excised and ligated into a circle, resulting in microdeletions at chromosomes. **b** Replication slippage model 2: ecDNA can be derived from replication slippage and recombination without deletion of original locus, leaving no chromosomal micro-gaps as they are filled by R-loop or homogenous recombination. This is more compatible when small regions of DNA containing one or a number of driver genes are selected. **c** Episome model: The ligation of DNA fragment pairs of inverted repeats in the replication bubble forms a single-strand circle when DNA replication is paused. **d** DSBs based model: in the process of DNA damage repair, DNA circle can be created by chromosomal rearrangements, which are mediated by the DNA damage repair mechanism non-homologous end-joining or microhomology-mediated end-joining. **e** Rolling circle model: Intrachromosomal recombination and "circularization" between tandem repeats produce circular molecules and shortened tandem sequences. **f** Translocation-deletion-amplification model: DNA segments derived from different chromosomes may form chimeric ecDNA sequences, which further invade and re-integrated into other chromosomes to generate novel DNA sequences

The biological source of ecDNA generation includes endogenous DNA damage (e.g. DNA replication stress), exogenous stress (e.g. carcinogens and pathogens) and aberrations in the DNA damage repair machinery [19]. Both ecDNA and homogeneously staining regions (HSR) of chromosomes can be formed through gene transcription and dramatically increase the complexity and plasticity of the genome [20–22]. Nevertheless, the underlying mechanisms of ecDNA biogenesis are not fully understood. In addition to simple self-ligation after DNA breaks, ecDNA can also be generated in multiple other ways [23, 24], and several models have been proposed, such as the breakage-fusion-bridge cycle [25], translocation-deletion-amplification [26], episome [27] and chromothripsis [28] models. The diverse genome compositions of ecDNA in multiple cancers imply complex multiple-step procedures in its formation, including the generation of DNA fragments from DNA damage (e.g. double-strand breaks), tandem duplication [29], breakage-fusion-bridge cycle [30] and chromothripsis-mediated chromatin rearrangement [31]. Non-homologous end-joining or microhomology-mediated end-joining repair mechanisms facilitate the rewiring of these DNA fragments in a random order, contributing to the generation of ecDNA [15, 32]. Importantly, ecDNA can self-replicate in the absence of tumor suppressors [1]. However, contradictory results have been reported regarding the contribution of DNA replication to ecDNA formation. One report showed that during replication, ecDNA can originate from loop excision and/or ligation of DNA fragments in the replication bubble where DNA replication is paused [32]. In contrast, other studies showed that inhibitors of DNA replication promoted the formation of ecDNA [33]. In addition, the DNA fragments released into peripheral blood by damaged cells in response to oxidative stress further contribute to ecDNA formation [34–36]. Collectively, the formation of ecDNA involves complex mechanisms (Fig. 1).

### Source sequences

**Fig. 2:**
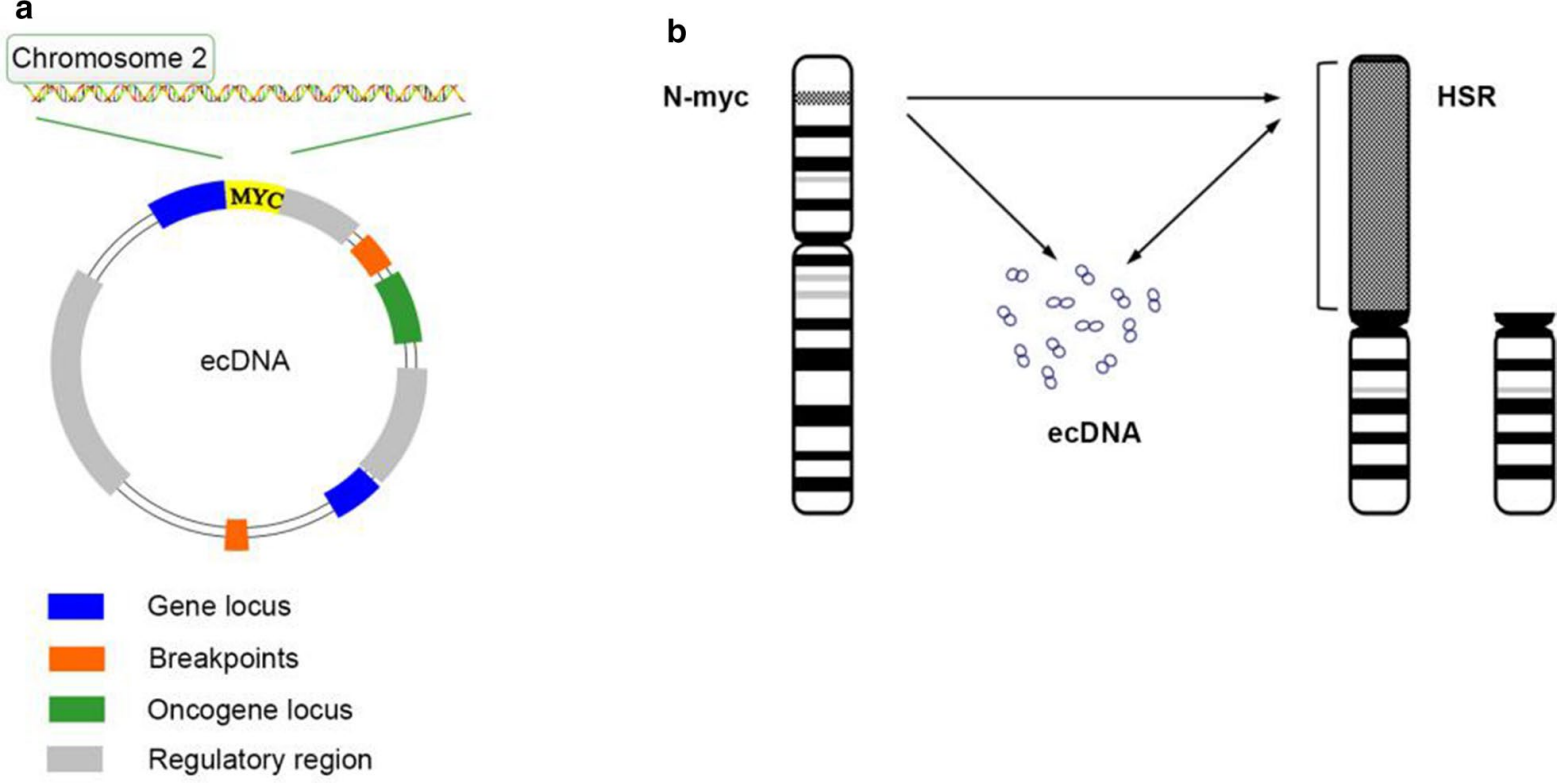
Gene amplification diagram of amplicons on ecDNA or HSR. **a** EcDNA carries not only complete genes, but also regulatory sequences such as promoters and enhancers. **b** The *N-MYC* amplification model. The *N-MYC* gene can be amplified either at ecDNA or at genome

Studies have shown the source sequences of ecDNA originate from multiple genomic sites from various individual chromosomes [23]. Storlazzi et al. demonstrated that ecDNA exhibits a high degree of heterogeneity in the sequence source, even within a single cell [37, 38]. Bioinformatic analyses of ecDNA sequences also indicate that oncogene amplification is unlikely to be the consequence of chromothripsis [23] (Fig. 2).

## The oncogenic functions of ecDNA

**Fig. 3:**
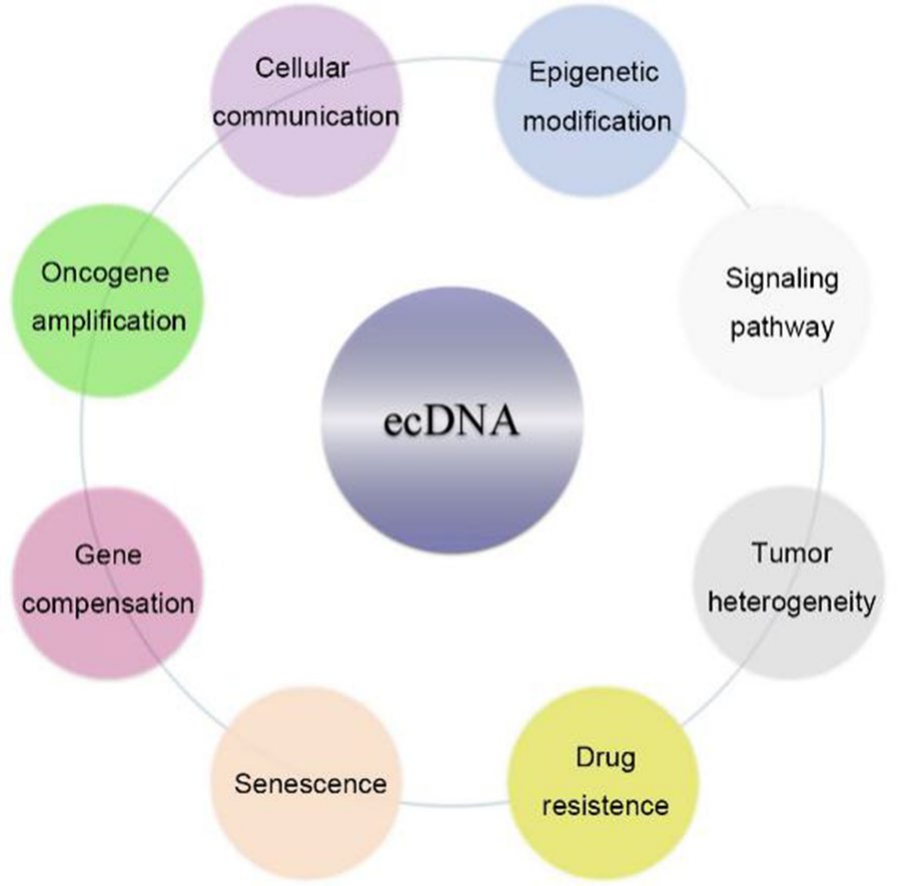
Overview of the oncogenic functions of ecDNA. EcDNA has a profound impact on multiple aspects of cancer phenotypes

Recent studies have demonstrated fundamental roles of ecDNA in cancer in modulating cell growth [15, 19, 39], metastasis/invasion [40, 41], autophagy [42, 43], DNA damage repair [34, 35], drug response [40, 44] and clinical outcome [15, 41] (Fig. 3). In addition, ecDNA contributes to intra-tumoral heterogeneity through genetic, epigenetic and microenvironmental factors [1, 2, 13, 18].

### Cell growth

The formation of ecDNA correlates with enhanced levels of DNA replication in highly proliferating cancer cells and exhibits survival benefits [2, 41]. In addition, ecDNA enhances the proliferation of cancer cells but suppresses the infiltration of immune cells, thus leading to an aggressive phenotype of elevated number of lymph nodes with micro-metastases in cancer patients [15].

### Intra-tumoral heterogeneity

Several studies showed that ecDNA increases the level of intra-tumoral heterogeneity in multiple cancer types [1, 2, 12]. The ecDNA originating from asymmetric chromatin segregation during mitosis and the massive amplification of oncogenes in ecDNA enable cancer cells to readily adapt to the evolving environment. Both primary and recurrent tumors show amplification of ecDNA-encoded genes (e.g. *MYC*, *MYCN*, *EGFR*, *PDGFRA* and *MET*), linking ecDNA to the evolvability of cancer cells under the selection pressure of the tumor microenvironment and therapeutic treatment [14]. In addition, as ecDNA is much more abundant in progressive tumors whereas high levels of HSR are more frequently observed in tumors under environmental stress, the balance between ecDNA and HSR of chromosomes is considered essential to determine the evolvability of cancer cells [18, 45].

### Autophagy

Several reports showed that ecDNA activates pathogen recognition receptors such as Toll-like receptor (TLR) family proteins, leading to inhibition of apoptosis and promotion of autophagy [42, 44]. In line with these results, some studies showed that ecDNA-containing cell-free DNA may regulate autophagy in a TLR9-dependent manner [42, 44]. Furthermore, studies in colon cancer cells have demonstrated that ecDNA transported in micronuclei or extracellular vesicles (EVs) can facilitate the induction of autophagy thus to promote cancer cell survival in response to chemotherapy [44].

### Drug sensitivity

Schimke et al. discovered that methotrexate resistance was attributed to *DHFR* gene amplification in ecDNA [22]. Meng et al. found that knock-down of *DHFR* resulted in increased sensitivity to methotrexate in DNA-PKcs-depleted ecDNA-containing cells but not in HSR-containing cells [46]. Glioblastoma cells have high levels of oncogenic *EGFRvIII* in ecDNA [18, 45]. Turner et al. performed structural analysis of *EGFRvIII* amplification on glioblastoma cells (GBM39) and found that ecDNA reintegrated into HSR during erlotinib treatment. Importantly, the ecDNA amplicon re-emerged when erlotinib was discontinued [18]. In addition, resistance to tyrosine kinase inhibitors of glioblastoma cells can be strengthened by adjustment of *EGFRvIII* levels in ecDNA [47]. These findings demonstrate that ecDNA modulates the drug sensitivity of cancer cells.

### Metastasis and invasiveness

Recent findings have linked ecDNA to increased cancer metastasis and poor patient outcomes. The overall level of ecDNA is markedly elevated in cancer patients with metastases than in patients without metastases [40, 41]. Mechanistically, ecDNA shuttle between the nucleus and cytoplasm and can be encapsulated in micronuclei or transported in EVs to cross the cell membrane or be exported to the extracellular space by exosomes [19, 48]. Cancer cells may use ecDNA as a messenger to transmit oncogenic information to other cell types in the microenvironment or to satellite tumors. ecDNA-mediated autocrine and paracrine signaling may result in increased invasiveness and chemoresistance and acquisition of the cancer stem cell–like phenotype [48]. In addition, ecDNA positively correlates with poor patient outcome [49]. The overall survival of patients carrying at least one circular amplicon of ecDNA was significantly poorer than that of patients without ecDNA-associated amplicons [15]. A meta-analysis of ecDNA measurement from 1076 patients with metastatic colorectal cancer confirmed a positive correlation between lower basal levels of ecDNA and better patient survival [41]. Furthermore, ecDNA can be used as a non-invasive prognostic tool that predicts the early relapse of thyroid cancer [50] and chemotherapy response in ovarian cancer [51].

### Senescence

Senescence is a potent barrier to prevent the malignant transformation of normal cells to cancer cells [52]. EcDNA functions as a reservoir of heterogeneous genetic material that endows cancer cells with rapid adaptation to environmental stress [53]. In yeast, the induction of senescence can be attributed to the accumulation of ecDNA with ribosome genes [54]. Importantly, daughter cells with less ecDNA exhibited a longer lifespan than mother cells with more ecDNA, and the ectopic expression of autonomously replicating sequence of ecDNA can trigger cell cycle arrest, cell death or age-related inflammation [54, 55]. However, the underlying mechanisms of how ecDNA circumvents the barrier of senescence to facilitate malignant transformation remain to be elucidated.

### Anti-tumor immunity

The elimination of ecDNA involves the entrapment of ecDNA within micronuclei, disappeared chromosomal γH2AX foci and the stimulation of immune responses [56]. Shimizu et al. found that ecDNA originating from anaphase chromosomes form micronuclei after hydroxyurea treatment [56]. Micronuclei facilitate the generation of aneuploid cells, which exhibit enhanced cell viability [57]. In neuroblastoma, ecDNA-containing micronuclei with amplified *MYCN* sequences were detected in vivo [58]. Notably, the DNA within micronuclei is prone to be released into the cytosol [59]. Dynamic for extracellular DNA interacts with micronuclei may be important for induction of anti-tumor immune response. Ji et al. found that downregulation of ecDNA-carried genes from colorectal and neuroectodermal tumor cells led to reduction of ecDNA genes by micronuclei expulsion which resulted in a decrease of tumor proliferation and malignancy [60]. As micronuclei are potential biomarkers for inflammation and DNA damage and known to trigger innate immune response including activation of cGAS-STING innate immune signaling [58], the cross-link of ecDNA and anti-tumor immunity is worth further investigation.

## The mechanistic actions of ecDNA

### Oncogene amplification

Oncogene amplification is one of the driving factors of tumorigenesis and can occur at either the HSR structures on chromosomes or ecDNA [61]. Studies have reported significantly elevated copy numbers of oncogenes encoded in ecDNA (e.g. *EGFR*, *MYC*, *CDK4*, and *MDM2*) [2]. The amplification of oncogenes in ecDNA markedly increases overall oncogene expression, which can be found in both primary and metastatic tumors regardless of treatment [18]. In addition to elevating oncogene levels by copy number amplification, ecDNA may re-integrate into HSRs of chromosome and/or affect DNA accessibility to further “stabilize” the expression of oncogenes (e.g. *EGFR* in glioblastoma) [47].

The distinct inheritance pattern of ecDNA differs from the traditional Mendel’s law of inheritance and raises the question of whether and how the location of amplified oncogenes impacts tumorigenesis. In this regard, Lobachev et al. found that the breaking sites of yeast chromosomes determine the consequences of gene amplification [62]. EcDNA is often observed to be produced from oncogene amplification, if the breaking sites locate between the hairpin break and the telomere. In contrast, when the break occurs between the oncogene and telomere, the amplification of oncogenes will generate HSR [62].

Importantly, a positive feedback regulatory loop between the elevated expression of ecDNA-encoded genes and the accumulation of ecDNA has emerged. Hull et al. found that yeast cells obtain high levels of ecDNA containing the copper resistance gene *CUP1* under copper exposure, and *CUP1* expression may cause further accumulation of *CUP1*-bearing ecDNA [63]. Moreover, Ji et al. showed that down-regulation of genes in ecDNA may result in the integration of ecDNA into cytoplasmic micronuclei and the subsequent reduction of ecDNA [60]. These results reveal a mechanistic link between the accumulation of ecDNA and oncogene hyper-activity.

### Chromosome rearrangements

As one of the major sources of somatic rearrangements, ecDNA exemplifies the mutagenic feature of the cancer genome [64]. Chromosomal rearrangements include translocations and/or insertions, which often result in oncogenes adjoining to transcriptional regulatory elements (e.g. promoters, enhancers) and the formation of fusion genes [65–67].

#### Rewiring enhancers

Morton et al. found that enhancers of *EGFR*, including endogenous enhancers as well as rewired enhancers from topological-associated domains, were co-amplified with oncogenes in glioblastoma [3]. These selectively skewed enhancers were also found in multiple cancer types (e.g. medulloblastoma, neuroblastoma and Wilms tumors) [3]. Helmsauer et al. further demonstrated that the majority of genomic rearrangement events involved ecDNA, challenging the current understanding of cancer genome remodeling [64].

#### Gene fusions

Gene fusions in ecDNA have been widely observed in leukemia and solid tumors, such as multiple myeloma [67], medulloblastoma [66] and gastric cancer [65]. Graux et al. identified a novel mechanism for the activation of tyrosine kinases in which the formation of ecDNA resulted in gene fusion between *NUP214* and *ABL1* in T-cell acute lymphoblastic leukemia [68]. Additionally, amplification of the *BCR*-*ABL1* fusion gene on ecDNA and the translocation of (9;22) (q34;q11) have been reported in chronic myeloid leukemia during imatinib treatment [69, 70]. Furthermore, L'Abbate et al. identified the *PVT1* gene on ecDNA as a hotspot for breakpoints whose amplification and rearrangements positively correlated with drug resistance and poor patient outcome in small cell lung cancer, indicating a crucial role of ecDNA in gene fusions [23].

### Epigenetic modifications

Epigenetic modifications, which includes the chemical modification of chromatin, gene compensation, chromatin interaction, and topological reconstruction, alter the accessibility of chromatin and ecDNA and play a key role in a variety of biological processes [2, 71, 72].

#### Histone modification

Previous studies showed that ecDNA are enriched with active rather than repressive histone markers [2]. Analyses of metaphase glioblastoma cells further demonstrated the high levels of active histone marks (H3K4me1, H3K27ac) on ecDNA, while the levels of repressive markers (H3K9me3, H3K27me3) were low [2].

#### Gene compensation

EcDNA plays a critical role in the compensation of histone genes. In *Saccharomyces cerevisiae*, a novel circular ecDNA with *HTA2-HTB2* amplification was generated to compensate for the effects of *HTA1-HTB1* deletion through the recombination between two Ty1 retrotransposon elements [72]. This finding suggests that loss of histone genes somehow activates a gene compensatory mechanism on ecDNA to maintain the proper expression levels of histone genes that are required for transcriptional activities.

#### Nucleosome accessibility

Topological studies have shown that ecDNA is packaged into circular chromatin and nucleosome units and lacks the canonical high-order of chromatin structure that is commonly seen in chromosomal DNA [2]. This unique structure of ecDNA leads to enhanced chromatin accessibility to transcriptional machineries to ecDNA-encoded genes [2].

#### Remote chromatin interaction

The circular chromosome conformation capture technology combining high-throughput sequencing (4C-seq) has been used to assess the chromatin connection on ecDNA [73]. Previous studies have shown that the remote interaction of active chromatin was enhanced via ecDNA, and even ultra-remote chromatin contact could be detected [2].

## Signaling pathways regulated by ecDNA

**Fig. 4:**
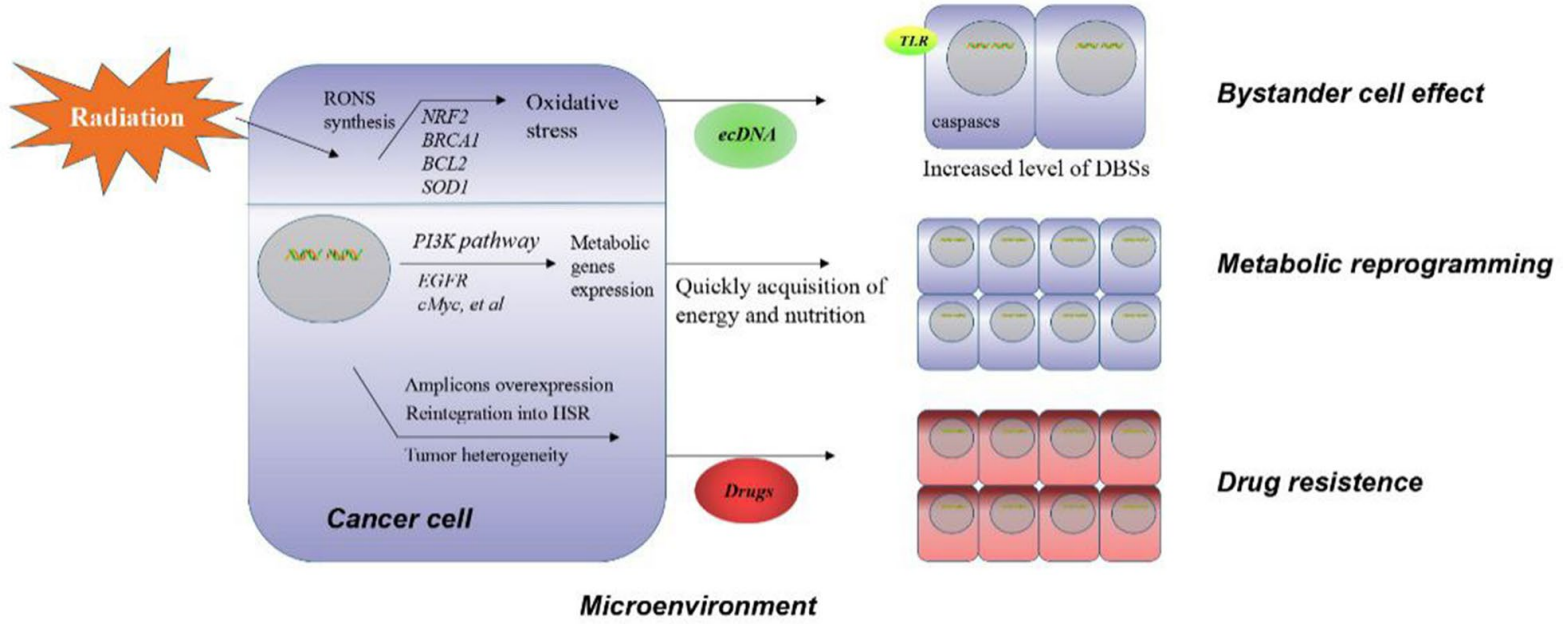
Mechanisms of ecDNA signaling in cancer. In response to oxidative stress, ecDNA induces the activation of inflammation, and to exert the bystander effect. Also, ecDNA regulates the metabolic reprogramming signaling to enhance the acquisition of energy in cancer cells

Better understanding of signaling pathways regulated by ecDNA is essential to elucidate the biological functions of ecDNA in cancer. These signals influence oxidative stress, inflammation and the bystander effect (Fig. 4) [34, 35, 74–76].

### Oxidative stress signaling and bystander effect

ecDNA signaling contributes to the development of adaptive responses and bystander effect under oxidative stress. Low dose ionizing radiation triggers oxidative stress, DNA modification, apoptosis, ecDNA generation and subsequent changes in bystander cells [35]. The damaged DNA in irradiated cells can be released into the intracellular space and received by bystander cells through caspase 3 and TLR (e.g. TLR9) dependent mechanisms [36, 74, 76]. Consistent with the responses of irradiated cells to oxidative stress, bystander cells also show alterations in nuclei shape, activation of nucleolar organizer regions, promotion of actin polymerization and elevation of double-strand break level (i.e. bystander effect) [76]. Accordingly, the increased level of ecDNA stimulates the rapid synthesis of reactive oxygen and nitrogen species, resulting in secondary oxidative stress and upregulation of anti-oxidant genes (e.g. *NRF2*, *KEAP1*, *SOD1*) [75].

### Pro-inflammatory signaling

The presence of ecDNA stimulates the production of pro-inflammatory cytokines that are deleterious to cancer cells [34]. Notably, ecDNA was shown to activate TLR9-MyD88-NF-kB signaling in the plasma of rheumatoid arthritis patients, leading to increases in pro-inflammatory cytokines (e.g. IL-6, TNF-α) [77]. In addition, the high GC content of ecDNA appears to affect the production of pro-inflammatory cytokines. One study reported that the GC-rich elements of ecDNA, but not genomic DNA, activated the production of IL-6 and TNF-α [77].

### Metabolic reprogramming signaling

High *EGFR* expression was shown to drive glycolysis through EGFR signaling, PI3K pathway and *c-MYC* dysregulation [78]. *EGFRvIII* signaling is stringently regulated in the metabolic events of glioblastoma. *EGFRvIII*-dependent metabolic reprogramming includes the synergistic regulation of fatty acid synthesis through Akt-SREBP1-dependent mechanisms [79] and the control of intra-tumoral cholesterol levels through LDLR-dependent signaling [80]. Importantly, *MYC* was shown to be co-amplified with *SQLE,* a key metabolic gene that encodes squalene monooxygenase in the sterol biosynthesis pathway [81]. Furthermore, *MYC* also upregulates *PYCRL*, a crucial regulator of ornithine to proline conversion, and its isoenzymes to enhance the synthesis of proline [82].

## ecDNA as a potential biomarker

An analysis of over 3200 clinical samples revealed that ecDNA was found in at least 14% of human cancers [2]. The frequency of ecDNA is likely to be higher in most aggressive cancer types, including glioblastoma, neuroblastoma, hepatocellular carcinoma, leukemia, lung and ovarian cancer [83]. Recent studies have revealed the potential utility of ecDNA in tumor diagnosis, prognosis and potential treatment of certain cancers on clinic [84, 85] (Table 2). The relationship of oncogenes amplified on ecDNA with drug sensitivity is also summarized below.

**Table 2:** The roles and potential applications of ecDNA in cancers

Cancers	Current advances	The connection between ecDNA and clinical applications	Refs.
I. Serving as potential biomarker to assess clinical outcomes			
Thyroid cancer	Development of a noninvasive diagnostic tool for biopsy	EcDNA is a component in liquid biopsy of thyroid cancer as a new plasma genotyping source	[42]
Cervical cancer	Development of a computational diagnosis method	The presence of ecDNA-viral structures is verified in cervical cancer samples	[43]
Ovarian cancer	Mouse xenograft model	Ciuculating DNA complements miRNAs and linear DNA for diagnosis	[44]
Non-small-cell lung cancer (NSCLC)	Application in the FLAURA phase III trial	Circulating tumor DNA (ctDNA) serves as primary objective to depict genetic tumor profile	[45]
Hepatocellular carcinoma (HCC)	Study in biopsy and plasma samples in HCC patients	ecDNA tracks real-time therapeutic responses and could overcome tumor heterogeneity	[98]

Cancers	Genes on ecDNA	Functions	Refs.
II. Elimination of oncogenes reside on ecDNA increases drug-sensitivity			
Glioblastoma	*MYC, EGFR, PDGFRα, ERBB2, CDK4, MDM2*	Amplification of *EGFRvIII* results in erlotinib resistance	[48]
Colon cancer	*DHFR, c-MYC*	Down-regulation of *DHFR* on ecDNA increases MTX sensitivity	[57, 94, 95]
Neuroblastoma	*MYCN*	Elimination of *MYCN* on ecDNA increases HU sensitivity	[67]
Cervical cancer	*DHFR*	Amplification of *DHFR* promote MTX resistance	[96]
Ovarian cancer	*MYCN, EIF5AR, CA125*	Decreased levels of ecDNA-form *CA125* after HU treatment	[97]
Breast cancer	*DHFR, HER2*	Loss of *HER2* residing on ecDNA has no effect on trastuzamab therapy	[98, 99]
Leukemia	*c-MYC*	Down-regulation of *c-MYC* promotes drug sensitivity	[100]
Oral squamous cell carcinoma	*MDR1*	Loss of *MDR1* enhances HU sensitivity	[101]

III. Extracellular vesicles carrying ecDNA transfer oncogenes and trigger tumorigenesis			
Ovarian cancer	Studies of EVs from cancer cells remain in the laboratory stage	ecDNA can be encapsulated in EVs. EVs might have applications on clinic for tumor diagnosis, prognosis or potential treatment	[19]

ecDNA may represent a novel tool for various clinical applications mainly in three aspects. First, ecDNA can be released into the peripheral blood system [86] and may serve as potential prognostic biomarkers of multiple cancers, such as thyroid cancer [50], cervical cancer [87], ovarian cancer [88] and non-small cell lung cancer [89]. As an example, ecDNA has been used in liquid biopsy of thyroid cancer as a new plasma genotyping source [50]. Second, elimination of oncogenes on ecDNA increases drug sensitivity [22, 69, 90, 47], providing a novel adjunctive therapeutic option for chemotherapy. Third, ecDNA-carrying EVs transport oncogenes and trigger tumorigenesis [19]. Thus, detecting and targeting EVs might have potential utility for cancer treatment.

## Conclusions and perspectives

Recent findings have revolutionized our understanding of ecDNA in cancer, highlighting the potential of ecDNA as a potential biomarker for personalized therapy. Since ecDNA is usually more stable than linear DNA, ecDNA may potentially be used in liquid biopsy [86]. However, the prognostic and/or diagnostic power of ecDNA remains undetermined. Clinical proof to support the feasibility of ecDNA as a biomarker is still lacking.

Despite the promising findings, several aspects of ecDNA remain to be elucidated. Studies have shown the possible origin and destination of ecDNA [91, 92]; however, the type of stress that initiates the generation of ecDNA and whether and how the ecDNA-encoded genes could be selectively induced under the evolving microenvironment remain unclear. ecDNA may influence bystander cells in response to oxidative stress, but whether the original ecDNA-producing cancer cells affect bystander cells to facilitate tumorigenesis and/or progression is still unknown.

In addition, the functions of ecDNA in multiple biological processes (e.g. cell development, aging, genomic instability, adaptive evolution, drug resistance, tumor development) also need to be further investigated. Elucidation of the underlying mechanisms of ecDNA may further shed light on cancer therapeutics.

## Data Availability

Not applicable.

## Abbreviations

HSR: Homogeneously staining region
BFB cycle: Breakage-fusion-bridge cycle
EGFR: Epidermal growth factor receptor
DHFR: Dihydrofolate reductase
LDLR: Low density lipoprotein receptors
SREBP1: Sterol regulatory element binding protein-1
PYCRL: Pyrroline-5-carboxylatereductase L
DSB: DNA double strand break
EV: Extracellular vesicle
